# Local Epidermal Growth Factor Receptor Signaling Mediates the Systemic Pathogenic Effects of *Staphylococcus aureus* Toxic Shock Syndrome

**DOI:** 10.1371/journal.pone.0158969

**Published:** 2016-07-14

**Authors:** Laura M. Breshears, Aaron N. Gillman, Christopher S. Stach, Patrick M. Schlievert, Marnie L. Peterson

**Affiliations:** 1 University of Minnesota, College of Pharmacy, Department of Experimental and Clinical Pharmacology, Minneapolis, Minnesota, United States of America; 2 University of Minnesota, College of Biological Sciences, Biotechnology Institute, Minneapolis, Minnesota, United States of America; 3 University of Iowa, Carver College of Medicine, Department of Microbiology, Iowa City, Iowa, United States of America; II Università di Napoli, ITALY

## Abstract

Secreted factors of *Staphylococcus aureus* can activate host signaling from the epidermal growth factor receptor (EGFR). The superantigen toxic shock syndrome toxin-1 (TSST-1) contributes to mucosal cytokine production through a disintegrin and metalloproteinase (ADAM)-mediated shedding of EGFR ligands and subsequent EGFR activation. The secreted hemolysin, α-toxin, can also induce EGFR signaling and directly interacts with ADAM10, a sheddase of EGFR ligands. The current work explores the role of EGFR signaling in menstrual toxic shock syndrome (mTSS), a disease mediated by TSST-1. The data presented show that TSST-1 and α-toxin induce ADAM- and EGFR-dependent cytokine production from human vaginal epithelial cells. TSST-1 and α-toxin also induce cytokine production from an *ex vivo* porcine vaginal mucosa (PVM) model. EGFR signaling is responsible for the majority of IL-8 production from PVM in response to secreted toxins and live *S*. *aureus*. Finally, data are presented demonstrating that inhibition of EGFR signaling with the EGFR-specific tyrosine kinase inhibitor AG1478 significantly increases survival in a rabbit model of mTSS. These data indicate that EGFR signaling is critical for progression of an *S*. *aureus* exotoxin-mediated disease and may represent an attractive host target for therapeutics.

## Introduction

The epidermal growth factor receptor (EGFR) is a single-pass receptor tyrosine kinase. In response to various stimuli, membrane bound sheddases of the ADAM family (a disintegrin and metalloproteinase) cleave EGFR ligands from the cell surface releasing them into the extracellular space allowing for autocrine and paracrine EGFR signaling [1]. Important EGFR ligands shed by ADAMs include transforming growth factor-α (TGF-α), amphiregulin (AREG), epidermal growth factor (EGF), and heparin-binding EGF-like growth factor (HB-EGF). Upon activation by shed ligands, EGFRs dimerize and auto- and trans-phosphorylate one another, forming docking sites for downstream intracellular effectors [2]. These effectors go on to regulate a number of critical cellular processes such as differentiation, survival, proliferation, adhesion, and migration. EGFR signaling is essential for proper epidermal barrier homeostasis and wound healing [3, 4]. During the inflammatory response to injury, EGFR signaling induces expression of toll-like receptors, antimicrobial peptides, cytokines, and chemokines from keratinocytes, contributing to the innate immune response to minimize the potential for infection and promote healing [5].

EGFR signaling also plays a role in the host response to a variety of pathogens, including *Staphylococcus aureus*. In *S*. *aureus* lung infections the gram-positive cell wall component, lipoteichoic acid (LTA) activates EGFR through ADAM-mediated shedding of HB-EGF, leading to mucin production in epithelial cells [6]. *S*. *aureus* protein A is a surface virulence factor that directly activates the EGFR leading to ectodomain shedding of molecules that modulate the immune response and potentiate bacterial invasion of epithelial tissues [7, 8]. The interaction of these surface molecules of *S*. *aureus* with the EGFR have only recently been investigated and are likely important during pathogenic processes.

Secreted *S*. *aureus* virulence factors also signal to the EGFR, though their mechanisms of action and downstream consequences are just beginning to be understood. The *S*. *aureus* superantigen (SAg) staphylococcal enterotoxin B (SEB) induces shedding of TGF-α leading to EGFR activation and crypt cell hyperplasia in human fetal small intestine explants [9]. Another SAg, toxic shock syndrome toxin-1 (TSST-1) induces ADAM17-mediated shedding of the EGFR ligands TGF-α and AREG from the surface of human vaginal epithelial cells (HVECs) leading to EGFR-dependent cytokine production [10]. The major *S*. *aureus* hemolysin, α-toxin, acts through the EGFR to induce proliferation of skin keratinocytes [11]. In this context, α-toxin may activate the EGFR through shedding of ligands via direct interaction with ADAM10, a surface sheddase and α-toxin receptor closely related to ADAM17 [12]. Recent work has also shown that α-toxin activates the EGFR in S9 airway epithelial cells and that this EGFR signaling is associated with the relative resistance of S9 cells to the cytotoxic effects of α-toxin [13]. These data demonstrate that ADAM-induced shedding of EGFR ligands and subsequent EGFR signaling are included in the host responses to *S*. *aureus* exotoxins. As *S*. *aureus* exotoxins are associated with severe diseases such as pneumonia, food poisoning, infective endocarditis, sepsis, atopic dermatitis and toxic shock syndrome (TSS) [14], the interaction of exotoxins with the EGFR warrants further investigation. The EGFR is an attractive host therapeutic target for infectious disease as EGFR inhibitors have been FDA-approved as cancer treatments and their clinical use is well documented [15, 16].

In the current study, the role of EGFR signaling in response to *S*. *aureus* exotoxins TSST-1 and α-toxin was explored in *in vitro*, *ex vivo*, and *in vivo* models. The SAg TSST-1 is the major cause of staphylococcal menstrual TSS (mTSS) and is associated with ~50% of non-menstrual TSS cases [14]. Human vaginal epithelial cells (HVECs) are used as an *in vitro* model to explore host signaling in response to TSST-1 and α-toxin [10, 17, 18]. The porcine vaginal mucosa (PVM) is remarkably similar in structure and physiology to its human counterpart and is used to model interactions of bacteria and bacterial products with the vaginal mucosa [17, 19–23]. Rabbits have been successfully used as an *in vivo* model of mTSS disease progression [22, 24, 25]. In one rabbit mTSS model, α-toxin enhances TSST-1 lethality at sub-lethal doses of both toxins [17] (Schlievert, unpublished observations) indicating that α-toxin may play a role in SAg-mediated disease. The establishment of these models makes the vaginal mucosa an ideal platform for the study of exotoxin/host interactions. It was hypothesized that like TSST-1, α-toxin induces cytokine production in HVECs through ADAMs and the EGFR. Furthermore, it was hypothesized that EGFR signaling is required for the *ex vivo* PVM cytokine response to TSST-1, α-toxin, and live *S*. *aureus* as well as disease progression *in vivo*. The data presented support these hypotheses, revealing the importance of local EGFR signaling during staphylococcal vaginal mucosal infections *in vivo*. The EGFR may therefore be an attractive host target for preventative and therapeutic measures against *S*. *aureus* infections.

## Results

### Alpha-toxin induces HVEC IL-8 production through ADAMs and the EGFR

**Fig 1:**
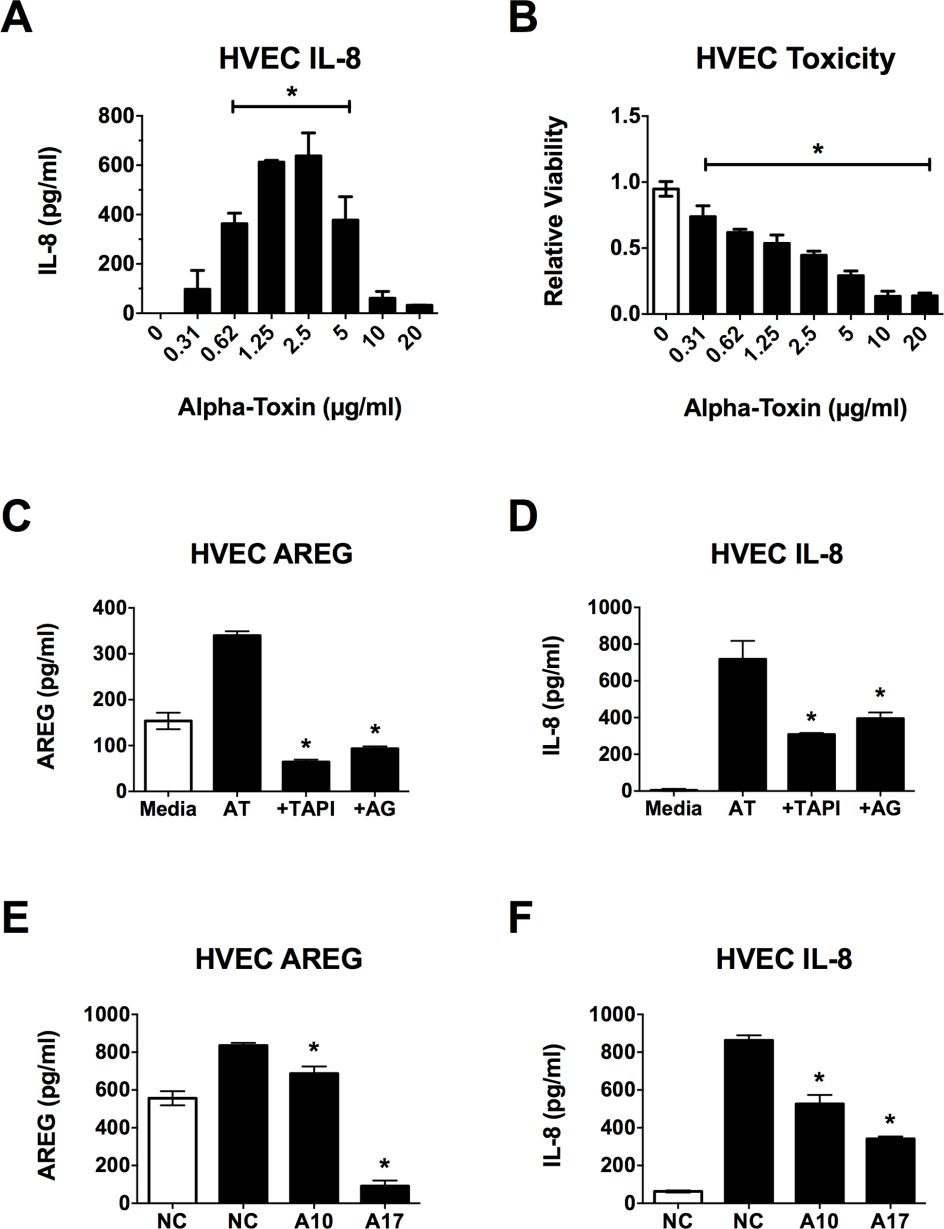
α-toxin induces AREG shedding and IL-8 production from HVECs. HVECs were exposed to α-toxin for 6 h and then processed for toxicity via MTT assay or IL-8 secretion and AREG shedding via ELISA. Where inhibitors were used, they were applied to HVECs 30 min prior to addition of α-toxin. (**A**) IL-8 secretion from and (**B**) viability of HVECs exposed to various doses of α-toxin. For both curves, the asterisks indicate doses showing significant differences from 0 (*p* < 0.0001). (**C**) AREG shedding and (**D**) IL-8 secretion in response to α-toxin is dampened in the presence of TAPI-1 and AG1478. White bars indicate media alone, black bars represent α-toxin treatment at 1 μg/ml. Asterisks indicate significant differences from α-toxin (AT) alone (*p* < 0.0001). (**E**) AREG shedding and (**F**) IL-8 secretion in response to α-toxin are dampened in ADAM10 (A10) and ADAM17 (A17) KD cell lines. White bars indicate media alone on negative control (NC) siRNA cells, black bars represent α-toxin treatment at 1 μg/ml. Asterisks indicate significant differences from α-toxin treated NC cells (*p* < 0.0001).

Analysis of EGFR ligand shedding was performed to investigate the potential role of ADAMs and the EGFR in the HVEC inflammatory response to α-toxin. While the dose of α-toxin chosen (1 μg/ml) produced some cell lysis (< 50%), it also consistently induced maximal, significant IL-8 production from HVECs over the 6 h course of the experiments (Fig 1A and 1B). A decline in IL-8 production was not observed until ~70% of cells were lysed during the 6 h incubation period. AREG, TGF-α, EGF and HB-EGF are potent activators of the EGFR [5]. Both AREG and TGF-α (but not EGF or HB-EGF) are shed by HVECs in response to TSST-1 [10]. While α-toxin did not induce shedding of TGF-α, EGF or HB-EGF from HVECs (data not shown), shedding of AREG was observed (Fig 1C). To investigate the roles of ADAMs and the EGFR in α-toxin-induced AREG shedding and IL-8 production, HVECs were treated with small molecule inhibitors prior to stimulation with α-toxin. The broad ADAM/MMP inhibitor TAPI-1 and the specific EGFR tyrosine kinase inhibitor AG1478 [26] reduced AREG shedding and IL-8 production in response to α-toxin with little cellular toxicity (Fig 1C and 1D, S1A Fig).

Because TAPI-1 is a pan-ADAM inhibitor, it cannot be used to discern which ADAMs mediate the epithelial α-toxin signal. ADAM10 is an epithelial cell receptor for α-toxin, and ADAM17 is the major sheddase for AREG making these two ADAMs likely targets of the observed α-toxin activity [1, 12]. ADAM10 and ADAM17 shRNA knock–down (KD) HVECs were used to determine if either of these sheddases was required for the HVEC response to α-toxin. Flow cytometry showed that ADAM10 KD was ~98% efficient while ADAM17 KD was ~48% efficient [10]. This level of ADAM17 KD in HVECs is sufficient to produce observable phenotypic responses to stimuli. Shedding of AREG in response to α-toxin was reduced by ~18% in the ADAM10 KD cells and ~89% in the ADAM17 KD cells (Fig 1E). It is unlikely that the observed increase of soluble AREG in response to α-toxin is from dead cells as it is reduced to levels significantly below background in the ADAM17 knockdown cell line, which would not affect full-length AREG released from dead or dying cells (Fig 1E). IL-8 secretion in response to α-toxin was reduced ~39% in the ADAM10 KD cells and ~60% in the ADAM17 KD cells (Fig 1F). These data suggest that both of these ADAMs are involved in the pro-inflammatory response to α-toxin, though (as is observed with TSST-1) ADAM17 plays a larger role in this response and is almost solely responsible for AREG shedding. Overall, the *in vitro* data demonstrate a clear role for EGFR signaling in the pro-inflammatory response to α-toxin through mechanisms similar to those previously observed for TSST-1.

### EGFR signaling is required for *ex vivo* production of IL-8 in response to TSST-1 and α-toxin

**Fig 2:**
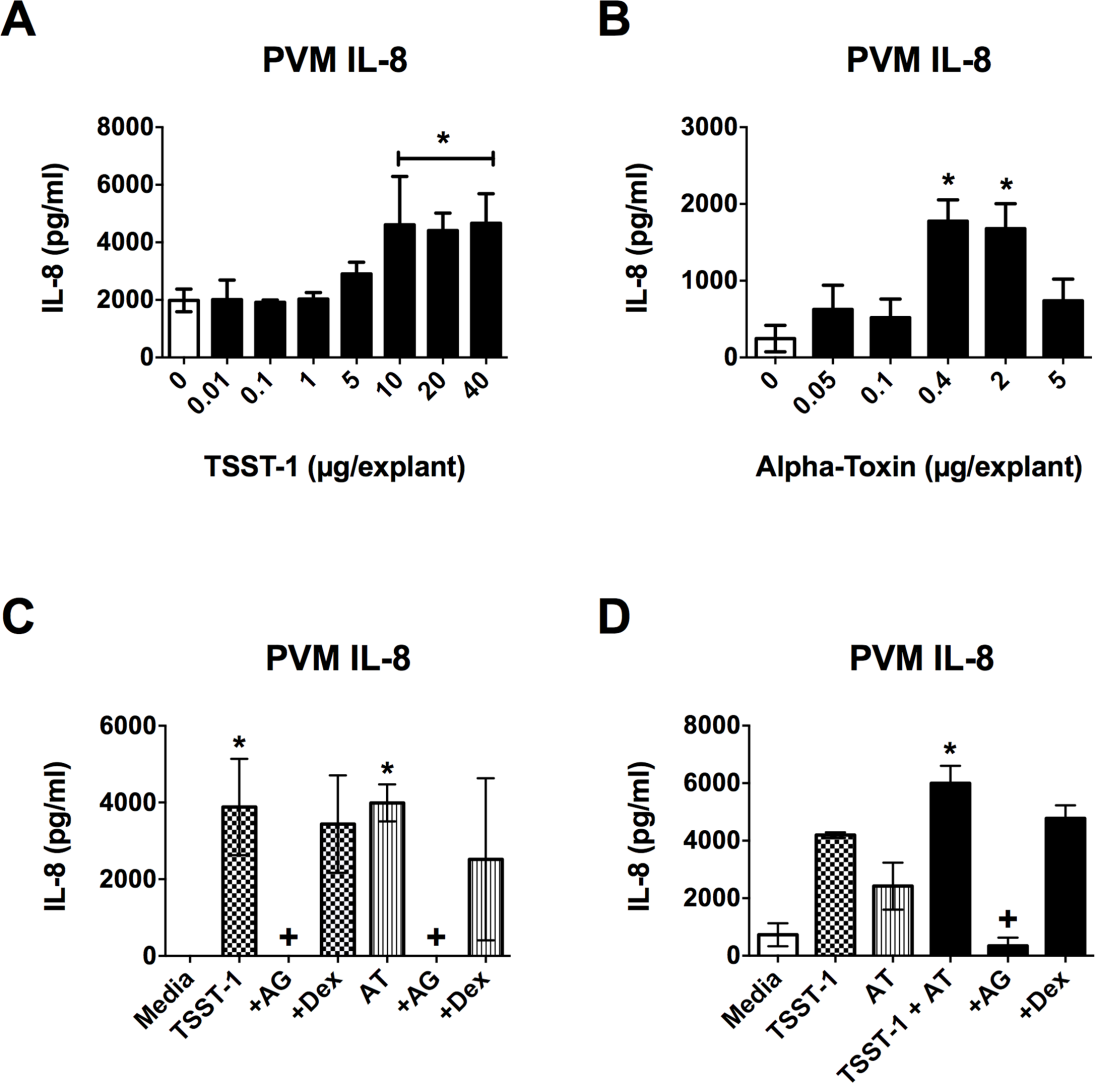
IL-8 production from PVM in response to TSST-1 and α-toxin is dependent on EGFR signaling. PVM explants were exposed to TSST-1 and/or α-toxin for 6 h and then processed for IL-8 production via ELISA. Where inhibitors were used, they were applied to explants 30 minutes prior to addition of toxin(s). IL-8 is produced in response to both (**A**) TSST-1 and (**B**) α-toxin in a dose-dependent manner. For both curves, the asterisks indicate doses showing significant differences from 0 (*p* < 0.0004). (**C**) IL-8 production in response to high doses of both TSST-1 (20 μg/explant) and α-toxin (AT) (2 μg/explant) is completely abrogated in the presence of AG1478 (AG– 40 μg/explant), but the dextrin vehicle (Dex– 10 μl of 15%) alone has no affect. Checkered bars represent TSST-1 treatment and striped bars represent AT treatment. Asterisks indicate significant differences from media alone, while crosses indicate significant differences from toxin alone (*p* < 0.0003). (**D**) Low doses of TSST-1 (5 μg/explant) and AT (25 ng/explant) have an additive effect on IL-8 production that is reduced to basal levels in the presence of AG1478 (4 μg/explant) with no dextrin vehicle effect (10 μl of 15%). White bars indicate media alone, checkered bars represent TSST-1 treatment, striped bars represent AT treatment, black bars represent TSST-1 + AT treatment. Asterisk indicates significant difference from media, TSST-1 and AT alone (*p* < 0.03), while cross indicates significant difference from TSST-1 + AT (*p* < 0.0009).

PVM was used to determine if EGFR signaling is required for the cytokine response to TSST-1 and α-toxin in an *ex vivo* tissue model. Both TSST-1 and α-toxin induced IL-8 production from PVM with TSST-1 exhibiting a maximal effect at ≥ 10 μg/explant (Fig 2A) and α-toxin exhibiting a maximal effect at 0.4–2.0 μg/explant (Fig 2B). The AG1478 EGFR inhibitor was used to determine if EGFR signaling is required for the pro-inflammatory response observed in PVM. IL-8 production in response to TSST-1, α-toxin or a combination of both was completely abrogated by EGFR inhibition (Fig 2C and 2D). The doses of toxins and AG1478 used were non-toxic to PVM indicating that any changes observed in IL-8 were not due to cell death (S1B–S1D Fig). It should also be noted that submaximal doses of TSST-1 and α-toxin have an additive effect on IL-8 production from PVM (Fig 2D) indicating that they are most likely acting through the same pathway to induce IL-8 production. These data demonstrate that EGFR signaling is required for the IL-8 response to TSST-1 and α-toxin in *ex vivo* vaginal tissue as is observed *in vitro* with HVECs.

### EGFR signaling mediates the global PVM IL-8 response to *S*. *aureus*

**Fig 3:**
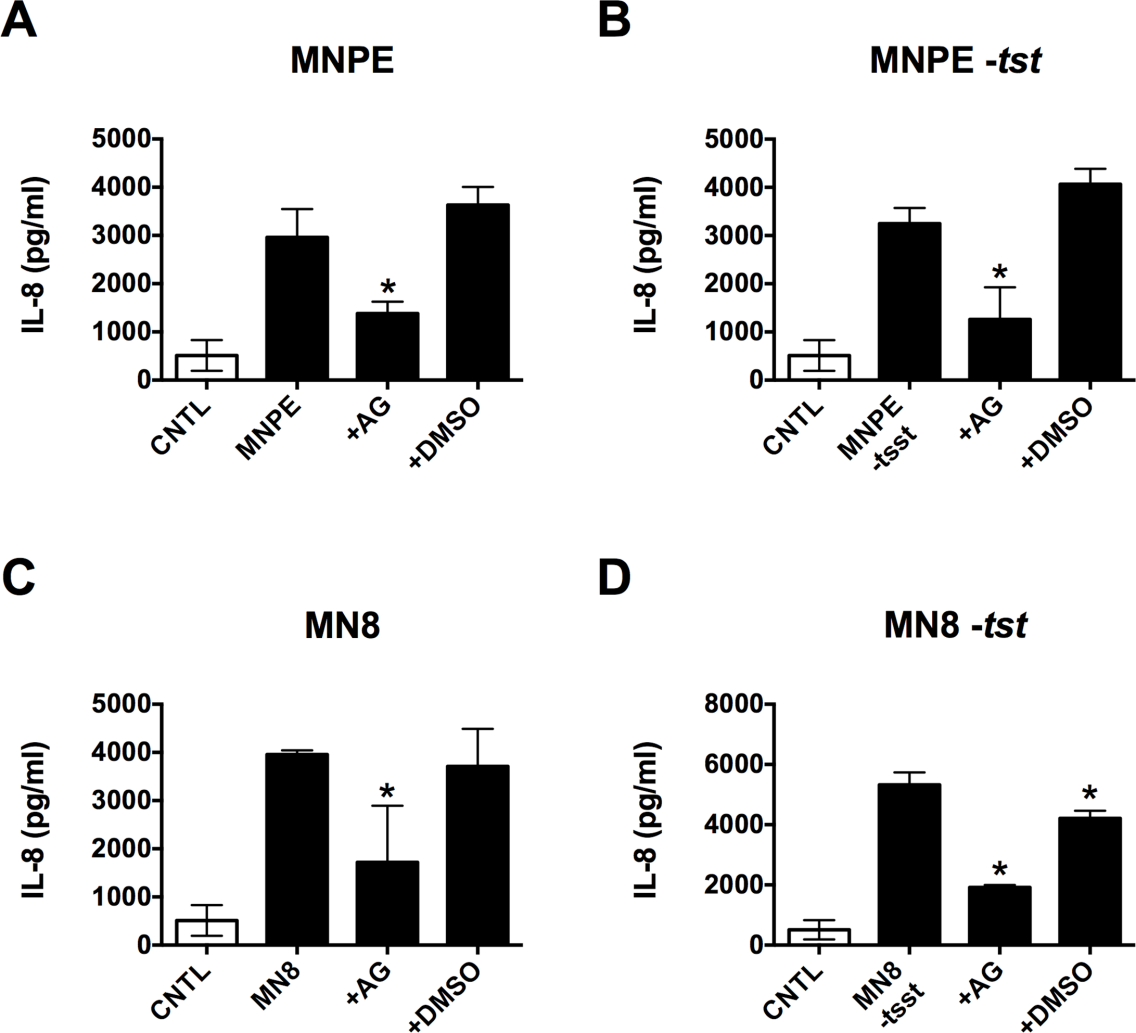
EGFR signaling mediates the PVM IL-8 response to *S*. *aureus*. PVM explants were inoculated with ~ 10^7^ CFU/explant and incubated for 6 h ± 4 μg/explant AG1478 (AG) or 4 μl/explant 10% DMSO vehicle prior to processing for IL-8 production (via ELISA) and CFU determination. (A-E) White bar, uninfected control (CNTL), black bars indicate presence of bacteria. Incubation of PVM with AG prior to infection with (**A**) MNPE, (**B**) MNPE *-tstH*, (**C**) MN8, or (**D**) MN8 *-tstH* significantly reduced IL-8 production (asterisks, *p* < 0.0002). DMSO alone had no effect.

To determine if EGFR signaling is required for the IL-8 response to *S*. *aureus*, multiple strains were tested in the PVM model. MNPE is a TSS^+^ strain that produces large amounts of TSST-1 and α-toxin when infecting the PVM [19, 21]. MNPE lacking TSST (-*tst*) was tested alongside the isogenic wild type (WT) strain. MN8 (a clinical isolate from the vagina of a woman with mTSS) does not produce full-length alpha toxin [21]. WT MN8 and a TSST knockout (*-tstH*) were also tested in the PVM model. To analyze the effect of EGFR signaling on IL-8 production in response to these organisms, the EGFR inhibitor AG1478 was applied to the PVM prior to infection. Production of IL-8 in response to every organism tested, regardless of which toxins were being produced, was reduced to near basal levels in the presence of AG1478 (Fig 3A–3D). All organisms were at ~ 10^7^ CFU/explant at the time of harvest and growth on the PVM tissue was unaffected by AG1478 (data not shown) as was tissue viability (S1D Fig), demonstrating that the observed reduction in inflammation was not due to a reduction in bacterial burden or host cell death in response to the inhibitor. These data show that EGFR-mediated IL-8 production induced by virulent *S*. *aureus* strains can be inhibited in the presence of live organism. They also indicate that IL-8 production is not dependent on the presence of TSST-1 or α-toxin, as we observed a similar increase in IL-8 from all strains in the absence of AG1478, even those not producing TSST-1 and/or α-toxin. This result is not unexpected since other *S*. *aureus* products such as protein A are known to activate ADAMs and the EGFR and to induce IL-8 production [6–8].

### EGFR signaling is required for mTSS progression *in vivo*

**Fig 4:**
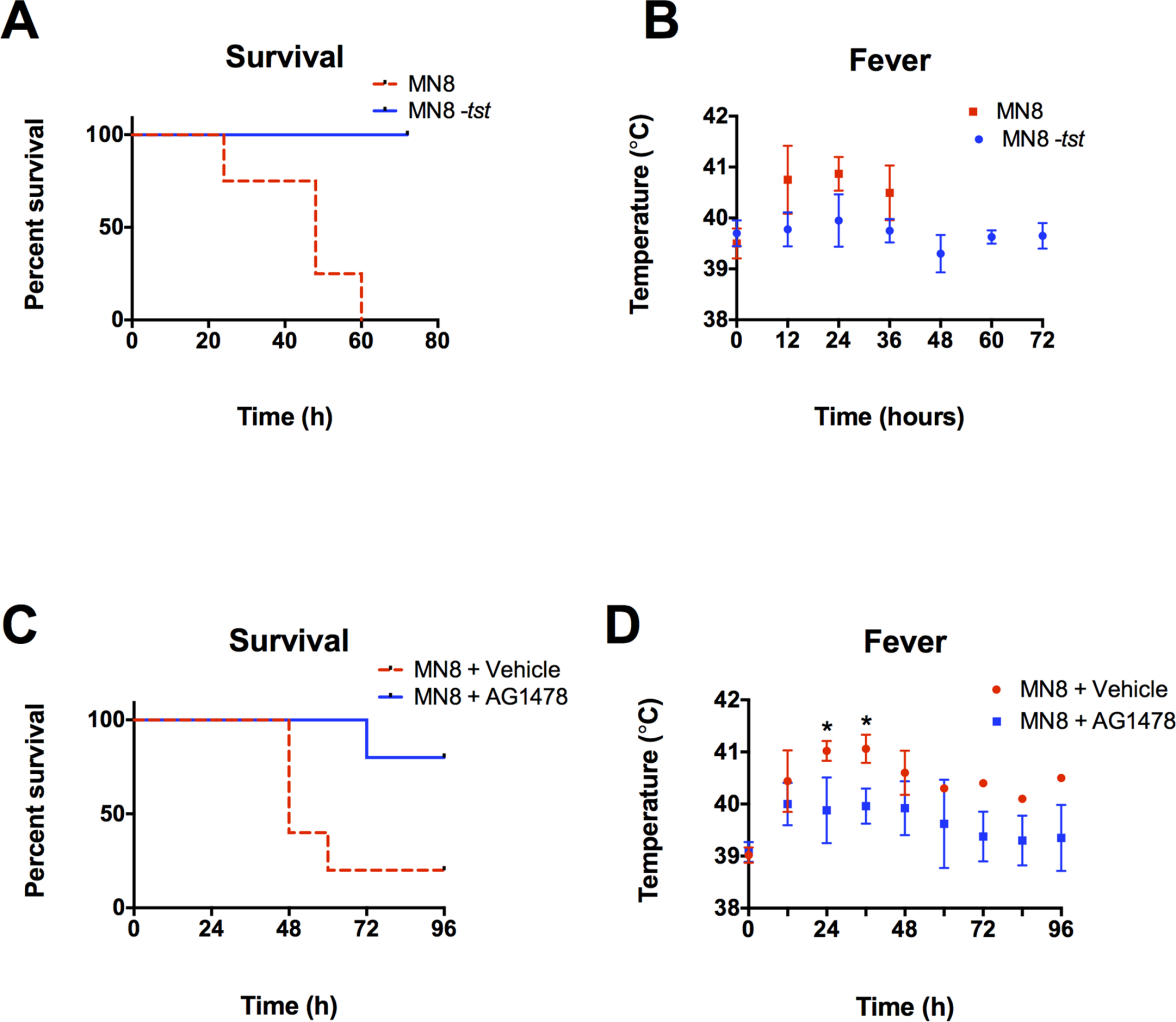
EGFR signaling is required for mTSS progression *in vivo*. (**A, B**) WT MN8 or MN8 *-tstH* were administered at 5 x 10^8^ twice daily for 3 days or until death, *N* = 1, n = 4. (**A**) Survival is significantly increased (*p* < 0.0069) and (**B**) fever is generally reduced in animals challenged with MN8 *-tstH* versus WT MN8. (**C, D**) In separate experiments, rabbits were intravaginally challenged with ~ 10^10^ MN8 + 8 mg/ml AG1478 or 30% beta cyclodextrin vehicle twice daily for 4 days or until death, *N* = 1, n = 5. (**C**) Survival is significantly increased in animals treated with AG1478 (*p* < 0.03). (**D**) Fever is generally decreased in animals treated with AG1478 reaching significance at 24 and 36 h, just prior to the death of the majority of infected, untreated animals (*p* < 0.005).

A rabbit model of mTSS was used to determine if lack of TSST production by *S*. *aureus* or inhibition of EGFR affects disease progression *in vivo*. Rabbits were challenged twice daily with WT MN8 or MN8 *-tstH*. Animals receiving MN8 *-tstH* exhibited 100% survival over 72 h, while those exposed to WT MN8 all died within 60 h of the initial administration (*p* < 0.0069, Fig 4A). The MN8 animals also showed consistently higher fevers at 12, 24, and 36 h (Fig 4B). In separate experiments, rabbits were challenged intravaginally twice daily with MN8 +/- AG1478 or the dextrin vehicle. Neither high single doses nor daily doses of AG1478 in dextrin are toxic to PVM tissue indicating they are safe for acute use in animals (S1E and S1F Fig). Four out of five rabbits inoculated intravaginally with MN8 + vehicle died within 60 h while four out of five rabbits receiving MN8 + AG1478 intravaginally survived the 96 h course of the experiment (*p* < 0.03, Fig 4C). Animals that received AG1478 also showed consistently reduced fever indicating that symptoms of systemic disease were attenuated in these animals (*p* < 0.005, Fig 4D). The time-line of disease progression observed in animals that died closely mimics that in humans presenting with mTSS [27]. These data demonstrate that loss of TSST-1 or inhibition of EGFR in the vaginal mucosa abrogates disease progression and significantly increases survival rates when animals are challenged with live *S*. *aureus*.

## Discussion

Secreted virulence factors such as SAgs and cytolysins contribute to many staphylococcal diseases [14, 28]. In fact, recent studies have found that vaccines that include these secreted factors are protective against lethal disease in animal models, highlighting their importance *in vivo* [29, 30]. The molecular mechanisms by which SAgs and cytolysins contribute to pathogenicity are beginning to be understood, allowing us to explore the use of therapies that target host molecules in combating *S*. *aureus*-mediated disease. The recent discovery that TSST-1 [10] and now α-toxin–two of the most pro-inflammatory *S*. *aureus* secreted toxins [21]–induce epithelial cytokine production through EGFR signaling makes this an attractive therapeutic pathway. The fact that there are already many EGFR inhibitors approved by the Food and Drug Administration for use in humans may ease the road to clinical development and implementation of EGFR inhibition for staphylococcal diseases.

The current work is to our knowledge the first evidence that inhibition of EGFR abrogates *S*. *aureus* disease progression. EGFR signaling plays a complex role in the inflammatory response of epithelial surfaces such as skin and mucosa to injury, allergy and infection [3, 5, 31–34]. A major consequence of this signaling is the epithelial production of cytokines leading to recruitment and local activation of immune cells such as neutrophils, macrophages, dendritic cells and lymphocytes. The data presented here reveals the importance of ADAM-mediated EGFR ligand shedding and subsequent EGFR signaling in the production of cytokines by epithelial cells in response to both TSST-1 and α-toxin.

Like TSST-1, α-toxin induces IL-8 in PVM in an EGFR-dependent manner. The current experiments did not explore the signaling pathway from EGFR activation to IL-8 production, but simply used IL-8 production as a read out of EGFR activity. It is likely that *il8* transcription is increased in response to α-toxin as activation of NF-κB and subsequent cytokine transcription is a common response to EGFR activation [35, 36]. Indeed, TSST-1 induces IL-8 production through the EGFR on the level of transcription [10]. The mechanism of inhibition of AG1478 is specific for the EGFR tyrosine kinase activity [26] indicating that this activity is required for the IL-8 response of both HVECs and PVM to α-toxin. We did observe cytotoxicity in response to α-toxin in HVECs and cannot rule out the possibility that the release of products (such as damage-associated molecular patterns) from dead cells may affect the shedding of AREG and/or release of IL-8 from neighboring intact cells in response to α-toxin. Contrary to this hypothesis, the doses of α-toxin eliciting an inflammatory response were not cytotoxic to the PVM, indicating direct signaling events between α-toxin and the EGFR of live cells in this model, which more closely mimics the *in vivo* environment. This data is interesting in light of the fact that most vaginal isolates that cause mTSS and have been pulsed-field gel electrophoresis typed fall into the USA200 category of which >95% harbor a mutation in the α-toxin gene that reduces but does not completely eliminate production of α-toxin [14, 21]. The low levels of α-toxin produced by these strains (such as the MN8 strain used for the *in vivo* studies) may contribute to disease through modulation of host cell signaling (such as the EGFR pathway) as opposed to cell lysis. It will be interesting to determine if/how EGFR inhibition affects disease outcome from mucosal infections with high α-toxin producers such as USA100 or USA300 strains.

Though both TSST-1 and α-toxin contribute to EGFR-induced IL-8 production, the current data show that they are not required for this activity. *S*. *aureus* strains lacking one or both toxins still induced equivalent levels of IL-8 in an EGFR-dependent manner. This is not surprising given the evidence that *S*. *aureus* cell wall components and surface virulence factors also stimulate EGFR signaling in epithelial tissues [6–8]. What is striking is the near complete abrogation of *in vivo* disease progression observed when EGFR is inhibited in the mTSS model. Lack of TSST-1 production or lack of EGFR activation provide similar protective outcomes indicating that in the case of mTSS (and perhaps other SAg-mediated diseases) EGFR signaling is required for disease, regardless of how it is activated. It remains to be seen if EGFR signaling mediates diseases such as *S*. *aureus* pneumonia in which α-toxin plays a major role through its interactions with ADAM10 [37]. At present, direct evidence for the consequence(s) of EGFR inhibition in the context of a mucosal bacterial infection is lacking. Because all pathogenic strains of *S*. *aureus* produce SAgs [14] and surface factors that activate EGFR signaling, inhibition of EGFR may help to prevent and/or treat mucosal *S*. *aureus* infections such as mTSS, non-menstrual TSS, and postrespiratory viral and necrotizing pneumonias.

The striking ability of an EGFR inhibitor to increase survival in the rabbit mTSS model leads one to ask how inhibition of the EGFR abrogates SAg-mediated disease. One possibility is that EGFR inhibition changes the local immune infiltrate in a way that halts mTSS progression. Inhibition of EGFR significantly reduces an array of cytokine expression and leukocyte infiltration of tissues in the mouse ear and lung in response to nontypeable *Haemophilus influenzae* (NTHi) infection [38]. This demonstrates that loss of EGFR signaling changes the local immune infiltrate, reducing the inflammation that is a major complication of NTHi infection. A reduction in the infiltrate of neutrophils, monocytes and lymphocytes is observed in the lung of a sensitized mouse model in response to SEB [39], a SAg related to TSST-1. The current results demonstrate that production of IL-8 is greatly reduced in response to secreted toxins and live *S*. *aureus* in the presence of the EGFR inhibitor AG1478. IL-8 is a potent chemokine for neutrophils. *S*. *aureus* is able to survive in the neutrophil phagosome and induce neutrophil lysis, which releases tissue-damaging molecules into the infected site [40]. These activities of *S*. *aureus* contribute to disease through enhanced organism survival, prolonged inflammation and local tissue damage. It is likely that attenuation of IL-8 production through EGFR inhibition reduces the local neutrophil infiltrate, perhaps dampening the effects of neutrophil lysis and allowing for quicker resolution of the inflammatory response. Future work will include histological examination of the epithelial tissues of animals exposed to *S*. *aureus* in the presence and absence of EGFR inhibitors to gain insight into how the immune response may be affected in animals protected from disease.

Under what conditions might EGFR inhibitors be used as therapy against *S*. *aureus* diseases? The most likely clinical setting for the use of EGFR inhibitors is as an adjunct therapy to antibiotic use in treatment of mucosal infections such as mTSS, non-menstrual TSS or pneumonia through systemic administration. SAgs and α-toxin contribute to lethal pulmonary infections [14, 41] and administration of EGFR inhibitors could prevent illnesses such as ventilator-associated and post-respiratory viral pneumonias. Inhibition of EGFR in the early stages of *S*. aureus mucosal infections may prevent disease progression, allowing adequate time for antibiotics to eliminate the organism.

## Methods

### Toxin purification

The cytolysin α-toxin was purified as previously described [17]. Briefly, α-toxin was isolated from *S*. *aureus* strain MNJA grown in beef heart medium. The culture was precipitated with ethanol at 4°C. The precipitate was resolubilized in water, and α-toxin was purified by isoelectric focusing (IEF). IEF was conducted in two phases: the first phase used a pH gradient of 3.5 to 10, followed by a second phase using a pH gradient from 7 to 9. The isoelectric point of α-toxin is 8.5. Purity was confirmed by SDS- PAGE and quantified using the Bio-Rad protein assay (Bio-Rad, Hercules, CA, USA).

TSST-1 was purified from *S*. *aureus* by an established method using successive thin layer IEF [42, 43]. TSST-1 (1 mg/ml), thus prepared, contained no detectable LPS, as tested by Limulus assay (Sigma); nor were peptidoglycan, lipoteichoic acid, hemolysin, protease, or lipase (<1 part/10^6^) detectable by combinations of bioassay and SDS-PAGE. Comparable results were obtained with multiple batches of TSST-1.

#### Cell culture

HVECs were purchased from ATCC (catalog number CRL-2616) and were described previously [44]. Cells were maintained on tissue culture-treated flasks or plates at 37°C in 7% CO2 in keratinocyte serum-free medium (KSFM: GIBCO, 10724–011) with 0.2 ng/ml human recombinant EGF, 50 μg/ml bovine pituitary extract (GIBCO, 37000–015), 0.4 mM CaCl2, 25 IU/ml penicillin, 25 μg/ml streptomycin, 40 μg/ml gentamicin, and 2.5 μg/ml amphotericin B (GIBCO). This is referred to as complete medium. KSFM supplemented only with 0.4 mM CaCl2 is referred to as minimal medium. ADAM10 and ADAM17 knock–down lines were generated as described [10].

### HVEC cytokine, shedding, and cell survival assays

HVECs were seeded at 50,000 cells/well in 96-well plates in complete medium. For all experiments, after 24 h the medium was replaced with minimal medium. Twenty-four hours later cells were exposed to minimal medium with or without 1 μg/ml α-toxin for the indicated time, unless another dose is indicated. Supernatant fluids were then removed, and secreted IL-8 or shed ectodomains were measured via ELISA according to the manufacturer’s protocols; IL-8 and AREG ELISAs were purchased from R&D Systems (DY208, DY262). Where inhibitors were used, they were added at a 2X concentration in one-half the final volume for 30 min at 37°C prior to addition of one-half the final volume of minimal medium alone or 2X α-toxin/TSST-1. TAPI-1 (Enzo Life Sciences, BML-PI134-0001) was used at a final concentration of 50 μM, and AG1478 (Tocris, 1276) was used at 1 μM. Cell survival was determined using the Cell Proliferation Assay (Promega, G3580).

### Bacteria

The USA200 isolates MNPE, MNPE *-tstH*, MN8, and MN8 *-tstH* were used in this study. MNPE was isolated from a post-influenza pulmonary TSS case in 1987 and most likely originated from a skin source [45]. This strain produces wild-type α-toxin. MN8 is a typical menstrual vaginal TSS strain, isolated from a patient in the 1980s [25]. This organism has a stop codon in the α-toxin gene that reduces by 50–100 fold the amount of the toxin that is produced. To produce MNPE -*tstH* and MN8 *-tstH* DNA was isolated using the Qiagen DNeasy Blood & Tissue Kit according to the manufacturer’s protocol for gram-positive organisms. Plasmid DNA was isolated using the Qiagen QIAprep Spin Miniprep kit in accordance with manufacturer’s protocol. In-frame deletions of *tstH* were generated as described previously [46] using PCR products amplified with the following primers: 5’–AGACTGGTATAGTAGTGGGTCTG– 3’ and 5’–TGATGCTGCCATCTGTGTT– 3’. Deletions were introduced using allelic exchange with pJB38 [47] and verified by PCR and Western immunoblot. All strains are methicillin sensitive.

### *Ex vivo* porcine vaginal mucosa

Specimens of normal porcine vaginal mucosa were excised from animals at slaughter in the U of MN Andrew Boss Laboratory of Meat Science and transported to the laboratory to be processed as previously described [48]. The vaginal tissue is a by-product of the slaughter of animals for human consumption and therefore is Institutional Animal Care and Use Committee (IACUC) exempt. Tissue was utilized within 3 h of excision. The explants were placed mucosal side up on a PET track-etched 0.4 mm cell culture insert (BD Bioscience) in 6-well plates containing fresh serum- and antibiotic-free RPMI 1640 (Gibco). The mucosal surface was continually exposed to air. For each experiment, indicated amounts of TSST-1 or α-toxin were placed on the PVM explants for the indicated times (n = 3 explants/treatment). Where the AG1478 inhibitor (Tocris, 1276) was used, the indicated mass of inhibitor or corresponding volume of vehicle was placed on explants 30 min and again 10 min prior to addition of toxins. Treated explants were incubated for 6 h at 37°C with 7% CO2. For IL-8 quantification, explants were vortexed in 250 μl PBS for 4 min at max speed, the supernates cleared by centrifugation, and analyzed via ELISA (R&D Systems, DY535). The viability of PVM explants was determined using the Cell Growth Determination kit (Sigma, CDG1). This kit is widely used to determine *ex* vivo toxicity of topical treatments ([49] and references therein). We did observe toxicity to PVM at the highest dose of α-toxin tested indicating that this assay is sensitive enough to detect toxicity in our experimental system (S1C Fig). The effects of treatment on tissue viability are expressed relative to untreated tissue control.

For bacterial infection of PVM, explants were exposed to MNPE (~10^6^ CFU/explant) with or without 30 min prior exposure to AG1478 (1.4 μg/explant) or 10% DMSO vehicle (8 μl/explant) for 6 h at 37°C with 7% CO2. Explants were vortexed in 250 μl PBS for 4 min at max speed to release IL-8 for quantification via ELISA or bacteria for analysis of CFU/explant. Bacteria were plated on tryptic soy agar containing 5% sheep’s blood (BD Diagnostics, 221261) using a spiral plater (Biotek, Microbiology International).

### Animal studies

Rabbit research was performed with approval given by the University of Iowa Institutional Animal Care and Use Committee. The approved animal protocol number was 1106140 and then replaced after three years by new protocol number 4071100. Numbers of rabbits required for experimentation was determined by power analysis and past experience. Young adult female New Zealand white rabbits weighing ~ 2–5 kg were obtained from Bakkom Rabbitry (Red Wing, MN). The animals were anesthetized with ketamine (10 mg/kg) and xylazine (10 mg/kg) prior to catheter insertion into the vagina. All animals were monitored every 15 min for appropriate depth of anesthesia and appropriate breathing. Upon recovery, all rabbits were administered 0.05 mg/kg twice daily of buprenorphine for pain relief. WT MN8 or MN8 *-tstH* at 10^8^–10^10^ were administered intravaginally twice daily. The cotton plug from a 1 ml pipette was inserted as a tampon and left in the vagina. Where used, a solution of 250 μl of 30% beta cyclodextrin (Sigma-Aldrich, 332593) or 250 μl of AG1478 at 8 mg/ml in 30% beta cyclodextrin was administered vaginally and allowed to absorb for 5 minutes prior to addition of bacteria. Administration of vehicle or AG1478 and MN8 was repeated every 12 h for 96 h or until death. Fevers were determined at each time point using rectal thermometers. There were no unexpected deaths. Surviving animals at 72 or 96 h were euthanized with Euthasol.

### Ethics statement

All animal experiments were performed according to established National Institutes of Health laboratory animal care and use guidelines. Protocols were approved by the University of Iowa Institutional Animal Care and Use Committee (protocols 1106140 and 4071100). All efforts were made to minimize suffering.

### Statistical analysis

All data shown are representative of at least 3 independent experiments with at least 3 replicates (N ≥ 3, n ≥ 3) unless otherwise noted. Statistical differences were analyzed using One-Way ANOVA with either Dunnett’s or Bonferroni’s multiple comparison test. When only two groups were being compared, Student’s *t* test was used with the Holm-Sidak multiple comparison correction. Survival data were analyzed by the log-rank (Mantel-Cox) test. Differences for all tests were considered significant if *p* < 0.05.

## Supporting Information

S1 Fig Toxicity Assays. HVECs or PVM were exposed to various toxins (black bars), inhibitors or vehicles (gray bars) for 6 h (unless otherwise noted) prior to processing for toxicity. HVEC toxicity is expressed as the percentage of cell death observed, while PVM toxicity is expressed as viability relative to controls (no treatment, white bars). PVM toxicity assays for (**A**) TSST-1, (**B**) α-toxin, (**C**) AG1478 in dextrin, and (**D**) AG1478 in DMSO show no significant reduction in tissue viability. (**E**) PVM was treated once daily for 3 days with AG1478 (11 μg/explant) or dextrin (8 μl of 30%) and no tissue toxicity was observed. (**F**) HVECs exhibit very minimal (< 0.1%) toxicity in response to TAPI-1 (50 μM) or AG1478 (1 μM). Asterisk indicates significant difference from media alone (*p* < 0.04).
(TIF)
