# Supplementary figures and images for: Local Epidermal Growth Factor Receptor Signaling Mediates the Systemic Pathogenic Effects of Staphylococcus aureus Toxic Shock Syndrome
Source: PLoS One. 2016 Jul 14;11(7):e0158969. doi: 10.1371/journal.pone.0158969 (PMC4944920; doi:10.1371/journal.pone.0158969)

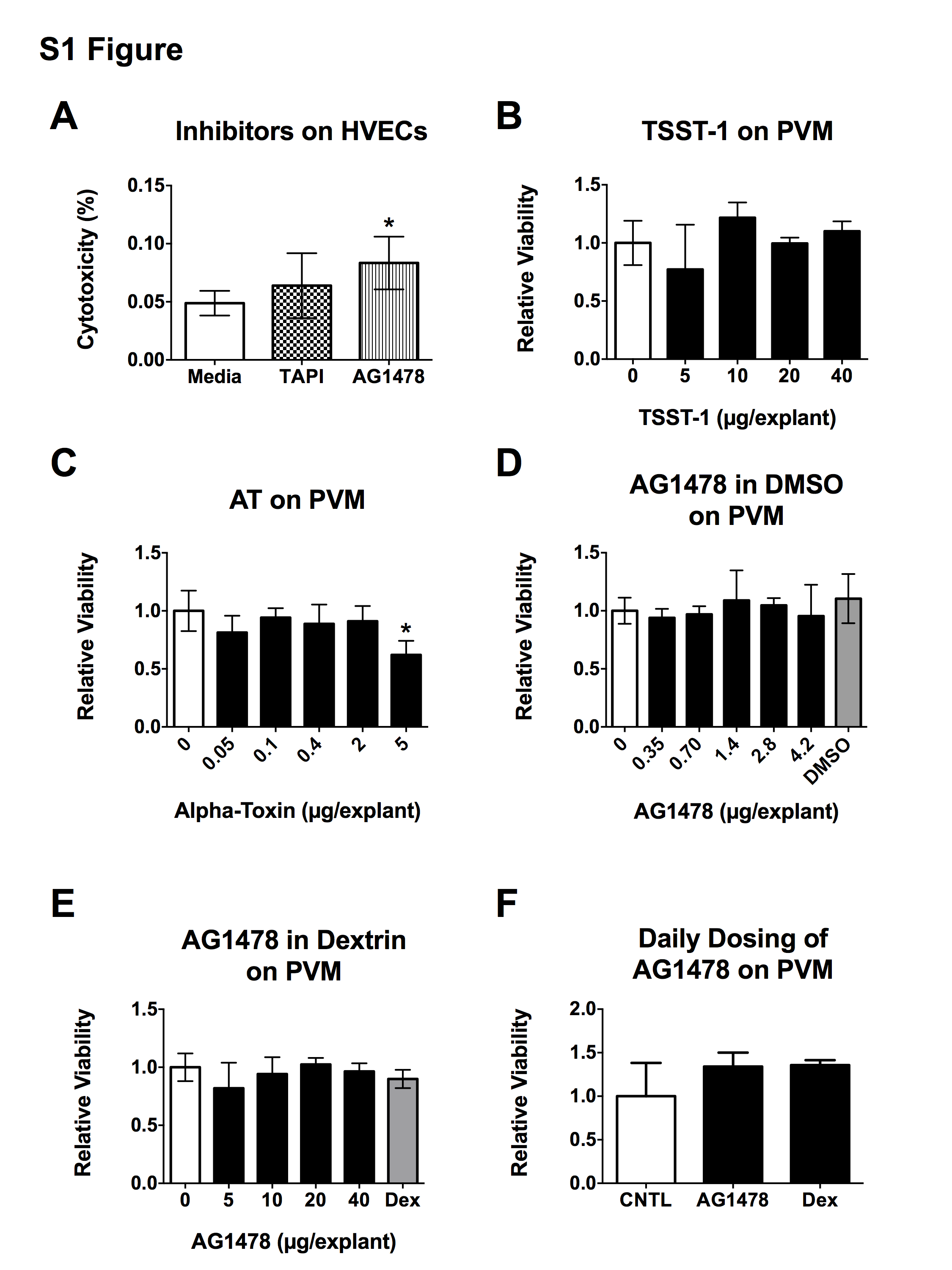

Supplement: S1 Fig — HVECs or PVM were exposed to various toxins (black bars), inhibitors or vehicles (gray bars) for 6 h (unless otherwise noted) prior to processing for toxicity. HVEC toxicity is expressed as the percentage of cell death observed, while PVM toxicity is expressed as viability relative to controls (no treatment, white bars). PVM toxicity assays for (A) TSST-1, (B) α-toxin, (C) AG1478 in dextrin, and (D) AG1478 in DMSO show no significant reduction in tissue viability. (E) PVM was treated once daily for 3 days with AG1478 (11 μg/explant) or dextrin (8 μl of 30%) and no tissue toxicity was observed. (F) HVECs exhibit very minimal (< 0.1%) toxicity in response to TAPI-1 (50 μM) or AG1478 (1 μM). Asterisk indicates significant difference from media alone (p < 0.04). (TIF) [file pone.0158969.s001.tif]
